# Anti-Müllerian hormone predicts positive sperm retrieval in men with idiopathic non-obstructive azoospermia—findings from a multi-centric cross-sectional study

**DOI:** 10.1093/humrep/dead125

**Published:** 2023-06-15

**Authors:** Edoardo Pozzi, Massimiliano Raffo, Fausto Negri, Luca Boeri, Antonino Saccà, Federico Belladelli, Simone Cilio, Eugenio Ventimiglia, Alessia d’Arma, Luca Pagliardini, Paola Viganò, Marina Pontillo, Roberta Lucianò, Maurizio Colecchia, Francesco Montorsi, Massimo Alfano, Andrea Salonia

**Affiliations:** Division of Experimental Oncology/Unit of Urology, URI, IRCCS Ospedale San Raffaele, Milan, Italy; Vita-Salute San Raffaele University, Milan, Italy; Division of Experimental Oncology/Unit of Urology, URI, IRCCS Ospedale San Raffaele, Milan, Italy; Urology Unit, ASST Spedali Civili di Brescia, Department of Medical and Surgical Specialties, Radiological Science and Public Health, University of Brescia, Brescia, Italy; Division of Experimental Oncology/Unit of Urology, URI, IRCCS Ospedale San Raffaele, Milan, Italy; Urology Unit, ASST Spedali Civili di Brescia, Department of Medical and Surgical Specialties, Radiological Science and Public Health, University of Brescia, Brescia, Italy; Department of Urology, Foundation IRCCS Ca’ Granda -Ospedale Maggiore Policlinico, University of Milan, Milan, Italy; Department of Urology, AO Papa Giovanni XXIII, Bergamo, Italy; Division of Experimental Oncology/Unit of Urology, URI, IRCCS Ospedale San Raffaele, Milan, Italy; Vita-Salute San Raffaele University, Milan, Italy; Division of Experimental Oncology/Unit of Urology, URI, IRCCS Ospedale San Raffaele, Milan, Italy; Urology Unit, Department of Neurosciences, Reproductive Sciences and Odontostomatology, University of Naples “Federico II”, Naples, Italy; Division of Experimental Oncology/Unit of Urology, URI, IRCCS Ospedale San Raffaele, Milan, Italy; Division of Experimental Oncology/Unit of Urology, URI, IRCCS Ospedale San Raffaele, Milan, Italy; Reproductive Sciences Laboratory, Obstetrics and Gynaecology Unit, IRCCS San Raffaele Scientific Institute, Milan, Italy; Infertility Unit, Foundation IRCCS Ca’ Granda -Ospedale Maggiore Policlinico, University of Milan, Milan, Italy; Laboratory Medicine Service, IRCCS Ospedale San Raffaele, Milan, Italy; Unit of Pathology, IRCCS Ospedale San Raffaele, Milan, Italy; Laboratory Medicine Service, IRCCS Ospedale San Raffaele, Milan, Italy; Unit of Pathology, IRCCS Ospedale San Raffaele, Milan, Italy; Division of Experimental Oncology/Unit of Urology, URI, IRCCS Ospedale San Raffaele, Milan, Italy; Vita-Salute San Raffaele University, Milan, Italy; Division of Experimental Oncology/Unit of Urology, URI, IRCCS Ospedale San Raffaele, Milan, Italy; Division of Experimental Oncology/Unit of Urology, URI, IRCCS Ospedale San Raffaele, Milan, Italy; Vita-Salute San Raffaele University, Milan, Italy

**Keywords:** anti-Müllerian hormone, idiopathic azoospermia, microsurgical testicular sperm extraction, sperm retrieval, testosterone

## Abstract

**STUDY QUESTION:**

Is it possible to identify a reliable marker of successful sperm retrieval (+SR) in men with idiopathic non-obstructive azoospermia (iNOA) undergoing microdissection testicular sperm extraction (mTESE)?

**SUMMARY ANSWER:**

A higher likelihood of +SR during mTESE is observed in men with iNOA and lower preoperative serum anti-Müllerian hormone (AMH) levels, with good predictive accuracy achieved using an AMH threshold of <4 ng/ml.

**WHAT IS KNOWN ALREADY:**

AMH has been previously linked to +SR in men with iNOA undergoing mTESE prior to ART.

**STUDY DESIGN, SIZE, DURATION:**

A multi-centre cross-sectional study was carried out with a cohort of 117 men with iNOA undergoing mTESE at three tertiary-referral centres.

**PARTICIPANTS/MATERIALS, SETTING, METHODS:**

Data from 117 consecutive white-European men with iNOA presenting for primary couple’s infertility associated with a pure male factor at three centres were analysed. Descriptive statistics was applied to compare patients with negative (−SR) versus +SR at mTESE. Multivariate logistic regression models were fitted to predict +SR at mTESE, after adjusting for possible confounders. Diagnostic accuracy of the factors associated with +SR was assessed. Decision curve analyses were used to display the clinical benefit.

**MAIN RESULTS AND THE ROLE OF CHANCE:**

Overall, 60 (51.3%) men had an −SR and 57 (48.7%) had a +SR at mTESE. Patients with +SR had lower levels of baseline AMH (*P* = 0.005) and higher levels of estradiol (E_2_) (*P* = 0.01). At multivariate logistic regression analysis, lower levels of AMH (odds ratio: 0.79; 95% CI: 0.64–0.93, *P* = 0.03) were associated with +SR at mTESE, after adjusting for possible confounders (e.g. age, mean testicular volume, FSH, and E_2_). A threshold of AMH <4 ng/ml achieved the highest accuracy for +SR at mTESE, with an AUC of 70.3% (95% CI: 59.8–80.7). Decision curve analysis displayed the net clinical benefit of using an AMH <4 ng/ml threshold.

**LIMITATIONS, REASONS FOR CAUTION:**

There is a need for external validation in even larger cohorts, across different centres and ethnicities. Systematic reviews and meta-analysis to provide high level of evidence are lacking in the context of AMH and SR rates in men with iNOA.

**WIDER IMPLICATIONS OF THE FINDINGS:**

Current findings suggest that slightly more than one in two men with iNOA had −SR at mTESE. Overall, men with iNOA with lower levels of AMH had a significantly higher percentage of successful SR at surgery. A threshold of <4 ng/ml for circulating AMH ensured satisfactory sensitivity, specificity, and positive predictive values in the context of +SR at mTESE.

**STUDY FUNDING/COMPETING INTEREST(S):**

This work was supported by voluntary donations from the Urological Research Institute (URI). All authors declare no conflict of interest.

**TRIAL REGISTRATION NUMBER:**

N/A

## Introduction

Azoospermia, defined as the absence of spermatozoa in the ejaculate, affects almost 1% of the male population and approximately 15% of infertile men (Minhas *et al.*, 2021). Of all azoospermic patients, 60% have an intrinsic testicular spermatogenic failure known as non-obstructive azoospermia (NOA) (Leslie *et al.*, 2022). Several genes and comorbid conditions have been linked with NOA (Olesen *et al.*, 2017; Azizi *et al.*, 2022; Wyrwoll *et al.*, 2022; Cannarella *et al.*, 2023). Although this holds true, a non-negligible proportion of patients suffer from NOA for which an identifiable and rationale aetiology cannot be found; these men are known to suffer from idiopathic NOA (iNOA) (Lee *et al.*, 2011). For this specific sub-set of men, testicular sperm extraction (TESE) surgery has emerged as the only available option to attempt and retrieve sperm for subsequent ART (Wosnitzer *et al.*, 2014; Salonia *et al.*, 2021; Schlegel *et al.*, 2021a,b). In this context, a number of techniques have been proposed, with conventional (cTESE) and microdissection TESE (mTESE) being the most popularized in terms of sperm retrieval (SR) rates and excisional damage minimization (Corona *et al.*, 2019; Esteves *et al.*, 2020; Salonia *et al.*, 2021). As such, several studies have reported heterogeneous data regarding positive SR rates (ranging from 30% to 60%) (Friedler *et al.*, 1997; Deruyver *et al.*, 2014; Corona *et al.*, 2019; Rohan *et al.*, 2021). Overall, the lack of clinically reliable biomarkers to predict positive SR at mTESE makes this procedure unnecessary for a substantial proportion of men with NOA (Ramasamy *et al.*, 2019; Tradewell *et al.*, 2022). Previous studies have theorized that anti-Müllerian hormone (AMH), a homodimeric glycoprotein of the transforming growth factor-β family, may effectively predict positive versus negative SR at mTESE in iNOA (Alfano *et al.*, 2017; Xu *et al.*, 2019; Song *et al.*, 2020; Benderradji *et al.*, 2021). AMH is secreted by Sertoli cells (SCs) during embryogenesis to ensure correct male sex differentiation, by causing the Müllerian ducts to regress (Toulis *et al.*, 2010). Moreover, as male puberty progresses and SCs become more mature, AMH levels drop significantly. As such, since AMH is exclusively produced by SCs in men, it has been proposed as an indirect marker of spermatogenesis itself, for SCs maturation (Rey *et al.*, 2000; Pierik *et al.*, 2003; Goulis *et al.*, 2009) and for immaturity of the testes stuck at the prepuberal stage (Alfano *et al.*, 2021). Although the predictive role of AMH has been theorized and demonstrated, most of the published studies rely on experience at a single centre with a limited number of men owing to the rarity of the condition itself. In this context, we sought to investigate and cross-validate the reliability of the prognostic role of preoperative circulating AMH to predict positive SR in a cohort of men with iNOA undergoing mTESE at three tertiary-referral andrology centres.

## Materials and methods

### Study cohort, variables, and outcome definition

The analyses of this multi-centre cross-sectional study were conducted on a cohort of 117 consecutive white-European men with iNOA presenting for infertility, which was defined as their partner not conceiving a pregnancy after at least 12 months of unprotected intercourse, according to the World Health Organization (WHO) criteria (WHO, 2018).

All patients underwent at least two consecutive semen analyses to confirm azoospermia and were then submitted to mTESE at three tertiary referral centres (IRCCS Ospedale San Raffaele, –Milan, Italy; Azienda Ospedaliera Papa Giovanni XXIII, Bergamo, Italy; and Fondazione IRCCS Ca'Granda Ospedale Maggiore Policlinico, Milan, Italy). iNOA was defined after exclusion of all known causes for NOA (Minhas *et al.*, 2021; Ventimiglia *et al.*, 2021). In this context, patients with the following clinical features were excluded from the study: azoospermic patients with testicular factors previously associated with infertility (cryptorchidism; grade II and III varicocele); genetic abnormalities previously associated with azoospermia, thus including mutations and polymorphisms of the cystic fibrosis transmembrane conductance regulator gene; homo and heterozygosis 1298 A > C for the Methylenetetrahydrofolatereductase gene; microdeletions on the Y chromosome; Klinefelter or Kallman syndromes; known hypothalamic/pituitary defects; either pituitary or testicular surgery and/or previous vasectomy; previous tumours, including testicular tumours; testosterone therapy; and any other known reason for genital tract obstruction. Patients were assessed by a thorough self-reported medical history, including age and comorbidities. Comorbidities were scored with the Charlson comorbidity index (CCI) (Salonia *et al.*, 2009). BMI, in kg/m^2^, was measured for each patient. Testicular volume (TV) was assessed using a Prader orchidometer (Boeri *et al.*, 2021). For the specific purpose of this study, we recorded the volume of each testicle and the mean value of the two sides. Data regarding the subsequent ART pathway, rates of viable pregnancies and live births after ART were collected for all patients with successful SR (+SR).

### Surgical technique and SR

All patients underwent mTESE at one of the three tertiary referral centres. mTESE was performed as detailed by Shlegel (1999). Whenever SR was negative (−SR) on one testicle, surgical exploration of the contralateral one was performed. At time of mTESE, the parenchyma was immediately placed in 5 ml of Quinn’sTM Sperm Washing Medium (Origio, Måløv, Norway) and minced mechanically with sterile slides. The sample was then transferred into a Falcon tube and centrifuged at 600*g* for 10 min at room temperature. The pellet was suspended in a minimum volume of 0.5 ml Quinn’sTM Sperm Washing Medium. SR was checked under an inverted microscope at ×400 magnification. Sperm counting was performed, and SR was expressed as the number of sperm/high power field (HPF) and then eventually cryopreserved. A +SR result was defined as the successful retrieval of at least one spermatozoon per 100 HPF (1 spz/100 HPF) as determined by experienced biologists. Data on sperm motility and vitality (as assessed by the swelling test) were also gathered before sperm cryopreservation in all patients.

### Histopathological analysis

A comprehensive histological analysis of all testicular specimens was performed. To obtain the final histopathological report, a testicular biopsy was performed during mTESE and sent for examination. All tissue samples were fixed in Bouin’s solution and formalin, and subsequently stained with haematoxylin–eosin. The findings were analysed based on the criteria proposed previously (McLachlan *et al.*, 2007). For the purpose of the study, to ensure consistent and uniform reporting of histopathological data, final histology was classified as: no germ cells; complete maturation arrest; incomplete maturation arrest; and normal parenchyma. Finally, we performed a histological classification of human spermatogenesis using the system developed by Johnsen (1970).

### Blood parameters and hormone levels

Venous blood samples were drawn from each patient between 7 a.m. and 11 a.m. after an overnight fast. FSH, LH, prolactin (PRL), thyroid-stimulating hormone (TSH), and 17b-oestradiol (E_2_) were measured in serum using a heterogeneous competitive magnetic separation assay. Inhibin B (InhB) and AMH were measured with an ELISA. Total testosterone (tT) levels were measured via a direct chemiluminescence immunoassay, and sex hormone-binding globulin (SHBG) levels were measured via a solid-phase chemiluminescent immunometric assay. The neutrophil–lymphocyte ratio (NLR) was measured. All blood analyses were performed in the same laboratory (IRCCS Ospedale San Raffaele).

### Statistical analysis

The statistical analyses consisted of several steps: first, patients were segregated according to SR (positive versus negative SR) at mTESE. Medians and interquartile ranges (IQR) or frequencies and proportions were reported for continuous or categorical variables, respectively. The Mann–Whitney and the Chi-square tests were used to compare the statistical significance of differences in the distribution of continuous or categorical variables among patients with +SR and −SR, respectively. Univariable (UVA) and multivariable (MVA) logistic regression models were fitted to predict the risk of +SR at baseline. Exploratory univariable analyses were initially performed with all variables. The MVA model was built by considering potential confounders. Decision curve analyses was used to display and test the clinical benefit of the identified factor associated with +SR at mTESE (Vickers and Elkin, 2006). Factors considered clinically associated with +SR at mTESE were assessed for diagnostic accuracy (sensitivity (SENS), specificity (SPEC), positive predictive value (PPV), negative predictive value (NPV) and AUC). Youden’s index calculation and AUC were used to identify the best cut-off to estimate the best SENS, SPEC, PPV, and NPV of the considered variables. All statistical tests were two-sided with a significance value set at 0.05. The analyses were conducted using R (2019), a language and environment for statistical computing (R Foundation for Statistical Computing, Vienna, Austria and GraphPad Software 7, San Diego, CA, USA).

### Study approval

Data collection followed the principles outlined in the Declaration of Helsinki; all patients signed an informed consent agreeing to provide their own anonymous information and tissue specimens for future studies. The study was approved by the Institutional Review Board (Authorization Protocol URI001-2010, further amended on December 2015 by the Ethic Committee IRCCS Ospedale San Raffaele, Milan, Italy).

## Results

**Figure 1.**
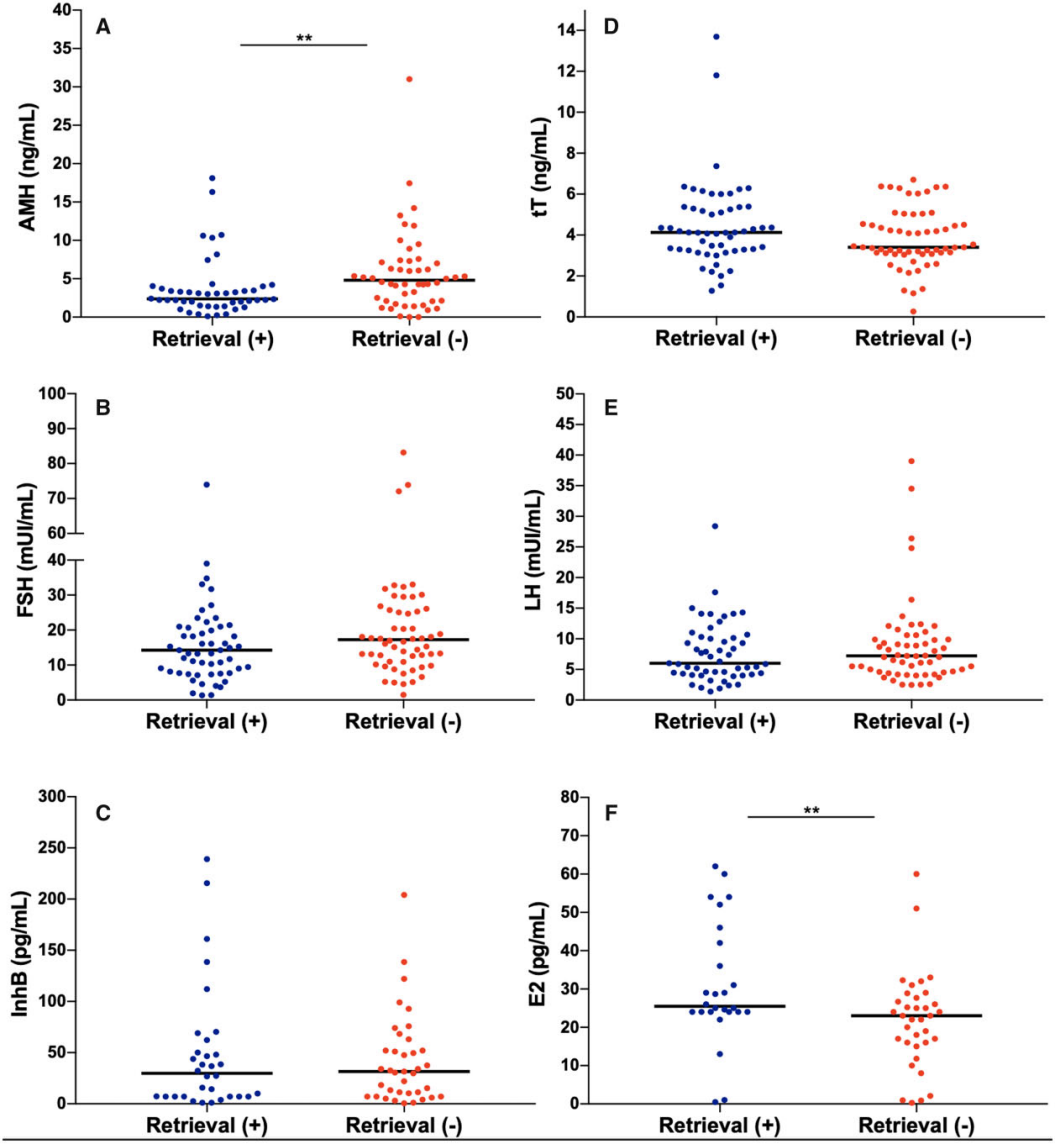
**Dot plots displaying the differences in hormonal levels between patients with iNOA with negative versus positive sperm retrieval at microdissection testicular sperm extraction.** Data analysed using Mann–Whitney test. iNOA, idiopathic non-obstructive azoospermia; AMH, Anti-Müllerian Hormone; InhB, Inhibin B; E2, 17β-oestradiol; tT, total testosterone.

Descriptive characteristics for the 117 patients with iNOA who underwent mTESE are listed in Table 1. Overall, 60 (51.3%) were −SR and 57 (48.7%) were +SR at surgery. Patients with +SR reported lower levels of baseline AMH (*P* = 0.005) and higher levels of E_2_ (*P* = 0.01). The +SR and −SR groups did not differ either in terms of other hormones tested (i.e. tT, FSH, LH, InhB, FSH/InhB, and PRL, Fig. 1) or of age, BMI, CCI ≥ 1, mean TV, smoking habit, semen volume, and NLR. Thirty (25.6%) patients had a normal parenchyma at the final histopathological report. Patients with a +SR reported higher rates of normal parenchyma (47.4% versus 5%, respectively, *P* < 0.001) compared to −SR men. In contrast, −SR patients displayed higher rates of no germ cell histology (46.7% versus 14%, *P* < 0.001) and of complete maturation arrest (40% versus 19.3%, *P* = 0.02) than +SR patients. A higher median Johnsen score was found in +SR men (6 versus 3, *P* < 0.001) as compared to −SR men. Moreover, when considering ART pathway initiation, 49 (86%) out of 57 +SR patients underwent ICSI while 8 (14%) had not initiated ART of any type at the last follow-up. Of patients undergoing ICSI, a viable pregnancy was achieved by 27 (55.1%) couples, and live births were obtained by 21 (77.7%) couples having started ART. Overall, a live birth from +SR was obtained in 21 (42.9%) out of 49 cases.

**Table 1.** Socio-demographic, clinical, and hormone characteristics of the study patients with idiopathic non-obstructive azoospermia.

Variable	Whole cohort	Sperm retrieval negative	Sperm retrieval positive	*P*-value
**Socio-demographic and clinical characteristics**				

Number of patients, No. (%)	117	60 (51.3)	57 (48.7)	<0.001
Age (years), median (IQR)	37 (33.3–40)	36.5 (33.8–40)	38 (33.3–40)	0.3
Partner age (years), median (IQR)	33 (31–36)	33 (31–36)	34 (32–37)	0.2
BMI (kg/m^2^), median (IQR)	25.3 (23.5–27.3)	24.5 (23.4–26.3)	26.2 (23.8–27.4)	0.2
Birthweight (g), median (IQR)	3500 (3102–4000)	3600 (3225–4025)	3500 (2800–3800)	0.1
Smoking, No. (%)				0.5
Yes/ex-smoker	31 (26.5)	18 (30)	13 (22.8)	
CCI, No. (%)				0.1
=1	2 (1.7)	0 (0)	2 (3.5)	
≥2	3 (2.6)	2 (3.3)	1 (1.8)	
Arterial hypertension, No. (%)	1 (0.9)	0 (0)	1 (1.8)	0.1
History of allergies, No. (%)	20 (17.1)	11 (18.3)	9 (15.8)	0.3
Mean testicular volume (Prader), median (IQR)	10.8 (8–12)	10.8 (8–12)	10.5 (9.3–13.1)	0.5
Infertility length (months), median (IQR)	18 (12–28.5)	18 (12–24)	18 (12–36)	0.4
Semen volume (ml)	3 (2.3–4)	2.9 (2–4)	3.5 (2.5–4.1)	0.4
Bilateral mTESE, No. (%)	68 (58.1)	58 (96.6)	10 (17.5)	<0.001

**Preoperative serum hormones**				

FSH (1.4–18.1 mUI/ml), median (IQR)	15.4 (9.6–23.5)	17.3 (11.4–26)	14.3 (7.9–20.9)	0.08
LH (1.7–8.6 mUI/ml), median (IQR)	7 (4.4–10.1)	7.2 (4.6–9.9)	6 (4.3–10.2)	0.4
tT (2.48–8.36 ng/ml), median (IQR)	4.1 (3.2–5.1)	3.4 (3.1–4.5)	4.1 (3.3–5.4)	0.1
SHBG (18.3–54.1 nmol/l), median (IQR)	31 (22.5–39.9)	31.9 (22.7–39.7)	31 (22.4–39.5)	0.9
Albumin (3.5–5 g/dl), median (IQR)	46.5 (45.3–48.2)	47.3 (46.2–48.7)	45.9 (45.2–47.1)	0.03
cfT, median (IQR)	0.06 (0.05–0.08)	0.06 (0.05–0.08)	0.06 (0.05–0.09)	0.8
E2 (<58 pg/ml), median (IQR)	24 (18–29)	23 (16–27.2)	25.5 (24–40.5)	0.01
InhB (25–325 pg/ml), median (IQR)	31.7 (7–52)	31.7 (10.3–52)	29.9 (7–53.1)	0.9
FSH/InhB, median (IQR)	0.5 (0.2–3)	0.5 (0.2–2.6)	0.6 (0.2–3.4)	0.8
AMH (0.77–14.5 ng/ml), median (IQR)	3.4 (2–6.1)	5 (2.1–7.1)	2.4 (1.6–3.7)	0.005
PRL (2.1–17.7 ng/ml), median (IQR)	12.5 (8–38.4)	12.7 (8.5–24)	10.2 (7.9–46.7)	0.9
TSH (0.25–5 μUI/ml), median (IQR)	2 (1.3–3.2)	1.6 (1.4–3.2)	2.1 (1.3–3.2)	0.5
Vitamin D (20–68 ng/ml), median (IQR)	23.6 (18.6–31.4)	22.8 (18.4–28.2)	24.5 (19–32)	0.5
Insulin (2.6–25 μU/ml), median (IQR)	7.3 (5.6–11.8)	7.7 (5.7–11.8)	7 (5.6–11.2)	0.9

**Preoperative blood parameters**				

Neutrophil–lymphocyte ratio, median (IQR)	2.3 (1.2–2.1)	1.4 (1.2–2.1)	1.9 (1.3–2.2)	0.3

**mTESE outcome**				

ART (any), No. (%)	49 (41.9)	0 (0)	49 (86)	<0.001
ICSI, No. (%)	49 (41.9)	0 (0)	49 (86)	<0.001
Viable pregnancy, No. (%)	27 (23.1)	0 (0)	27 (47.4)	<0.001
Live birth, No. (%)	21 (18)	0 (0)	21 (36.8)	<0.001

**Histology**				

No germ cells, No. (%)	36 (30.8)	28 (46.7)	8 (14)	<0.001
Complete maturation arrest, No. (%)	35 (29.9)	24 (40)	11 (19.3)	0.02
Incomplete maturation arrest, No. (%)	16 (13.7)	5 (8.3)	11 (19.3)	0.1
Normal parenchyma, No. (%)	30 (25.6)	3 (5)	27 (47.4)	<0.001
Johnsen score, median (IQR)	4 (1–8)	3 (1–4)	6 (4–8)	<0.001

IQR, interquartile range; CCI, Charlson Comorbidity Index; tT, total testosterone; SHBG, sex hormone-binding globulin; cFT, circulating free testosterone (calculated using the Vermeulen formula); InhB, Inhibin B; AMH, anti-Müllerian hormone; TSH, thyroid-stimulating hormone; E2, 17β-oestradiol; PRL, prolactin; Hb, haemoglobin; PCR, protein-C reactive; mTESE, microdissection testicular sperm extraction; Hormone reference values are reported. Medians and interquartile ranges (IQR) or frequencies and proportions were reported for continuous or categorical variables, respectively. The Mann–Whitney and the Chi-square tests were used to compare the statistical significance of differences in the distribution of continuous or categorical variables among patients with +SR and −SR, respectively.

Table 2 reports UVA and MVA logistic regression analyses. At MVA, lower levels of AMH (OR: 0.79; 95% CI: 0.64–0.93, *P* = 0.03) were associated with +SR at mTESE, after adjusting for possible confounders (age, mean TV, FSH, and E_2_). None of the other preoperative clinical parameters achieved statistical significance.

**Table 2.** Univariable and multivariable logistic regression analyses showing potential predictors of positive sperm retrieval at microdissection testicular sperm extraction among men with idiopathic non-obstructive azoospermia.

	UVA		MVA	
Variable	OR (95% CI)	*P*-value	OR (95% CI)	*P*-value
Age	1.04 (0.97–1.12)	0.3	1.06 (0.95–1.19)	0.4
Smoking	1.04 (0.97–1.12)	0.9	–	
BMI	1.03 (0.93–1.14)	0.6	–	
CCI ≥1	1.23 (0.47–3.21)	0.7	–	
Mean testicular volume (Prader)	1.04 (0.96–1.14)	0.4	1.09 (0.93–1.32)	0.3
History of cryptorchidism	1.18 (0.47–2.92)	0.7	–	
Infertility length	1.01 (0.99–1.03)	0.4	–	
AMH	0.89 (0.81–0.97)	0.04	0.79 (0.64–0.93)	0.03
AMH <4 ng/ml	5.67 (2.76–12.11)	0.0001	–	
AMH/tT	0.72 (0.51–0.92)	0.07	–	
tT	1.23 (1.03–1.54)	0.1	–	
SHBG	1.00 (0.98–1.03)	0.9	–	
FSH	0.97 (0.95–0.99)	0.1	1.01 (0.95–1.07)	0.7
LH	0.97 (0.91–1.02)	0.3	–	
InhB	1.00 (0.99–1.01)	0.6	–	
E2	1.05 (1.01–1.09)	0.02	1.04 (0.99–1.09)	0.1

CCI, Charlson Comorbidity Index; tT, total testosterone; InhB, inhibin B; AMH, anti-Müllerian hormone; E2, 17β-oestradiol; SHBG, sex hormone-binding globulin; UVA, univariable logistic regression analyses; MVA, multivariable logistic regression analyses.

**Figure 2.**
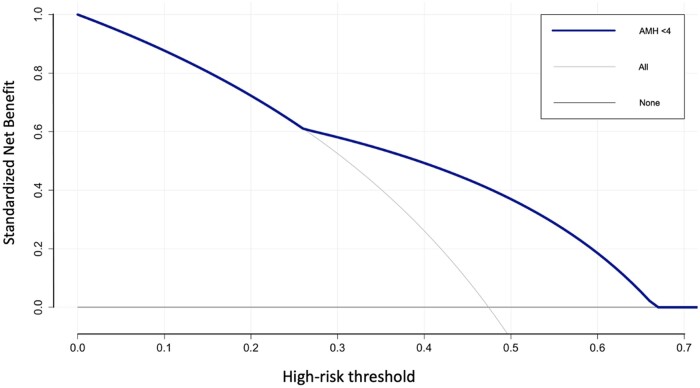
**Decision curve analysis displaying the superior net benefit of using AMH <4 ng/ml for positive sperm retrieval at mTESE in men with idiopathic non-obstructive azoospermia.** In decision curve analysis, the lines labelled ‘testing all’ and ‘testing none’ represent reference lines that help interpret the results of the analysis. These lines provide a benchmark against which the performance of a diagnostic or predictive model can be compare, allowing researchers, and decision-makers to evaluate the clinical utility and potential benefits of a test or intervention. The ‘testing all’ line represents a scenario where all individuals, regardless of their risk profile, are subjected to the test or intervention being evaluated. It assumes that the test has perfect accuracy and everyone benefits from it. In this scenario, the model’s net benefit is calculated by comparing the proportion of individuals who benefit from the test to those who are harmed. On the other hand, the ‘testing none’ line represents a scenario where no one undergoes the test or intervention. This line assumes that no one benefits from the test, and the net benefit is determined solely based on the proportion of individuals who are harmed by false positives or unnecessary interventions. AMH, Anti-Müllerian Hormone; mTESE, microdissection testicular sperm extraction.

Table 3 shows the diagnostic accuracy of the factors potentially considered clinically associated with +SR at mTESE, expressed in terms of SENS, SPEC, PPV, NPV, and AUC according to Youden’s index calculation. A cut-off of AMH (AMH <4 ng/ml) was identified to be the best in terms of SENS, SPEC, PPV, NPPV, and AUC for +SR at mTESE. The SENS, SPEC, PPV, and NPV of AMH <4 ng/ml were 73.9%, 66.6%, 66.6%, and 73.9%, respectively; the AUC was 70.3% (95% CI: 59.8–80.7). The AUC of AMH <4 ng/ml is graphically displayed in Fig. 2.

**Table 3.** Sensitivity, specificity, predictive values, and AUC of population clinical characteristics and outcome of microdissection testicular sperm extraction.

Variable	AUC (95% CI)	Sensitivity	Specificity	PPV	NPV
Age	56.4 (44.1–68.1)	21.4	88.4	60	58.2
Smoking	54.6 (0.4–0.7)	6.8	97.5	66.6	59.1
BMI	57.2 (0.5–0.7)	55	68.1	59.4	64
CCI ≥ 1	54.5 (39.8–69.3)	7.6	100	100	59.3
Mean testicular volume	54.3 (40–61.3)	94.1	18.4	50.8	77.7
History of cryptorchidism	54.5 (39.8–69.3)	76.9	100	100	59.3
Length of infertility	56.6 (41.1–72)	36	82.7	64.2	60
AMH	66.4 (55.5–77.3)	82.6	64.7	67.9	80.5
AMH < 4	70.3 (59.8–80.7)	73.9	66.6	66.6	73.9
AMH/tT	67.4 (56.6–78.2)	76	66.6	67.3	75.5
tT	58.7 (48.2–69.3)	66.6	54.2	57.1	64
SHBG	50.6 (38.5–62.6)	65.1	43.3	51.8	57.1
FSH	59.4 (48.9–69.9)	43.6	74.1	61.5	58.1
LH	54 (43.3–64.8)	45.2	68.9	57.1	57.9
InhB	48.9 (35.1–62.7)	46.8	59.4	50	56.4
E2	64.1 (54.1–81.9)	46.1	77.1	60	65.8

CCI, Charlson Comorbidity Index; AMH, anti-Müllerian Hormone; tT, total testosterone; SHBG, sex hormone-binding globulin; InhB, inhibin B; E2, 17β-oestradiol; PPV, positive predictive value; NPV, negative predictive value.

**Figure 3.**
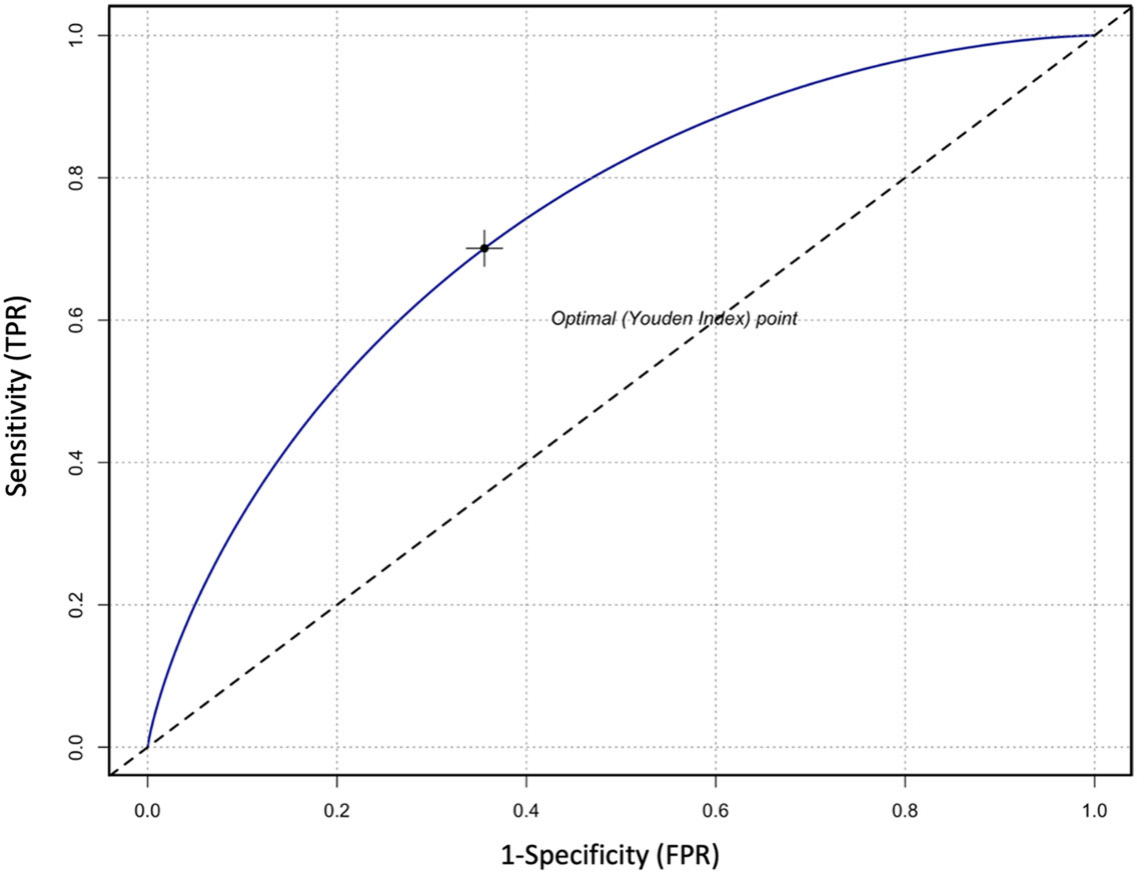
**Sensitivity and specificity of AMH <4 ng/ml for sperm retrieval in men with idiopathic non-obstructive azoospermia.** TPR, true positive rate; FPR: false positive rate; AMH: anti-Müllerian hormone.

Figure 3 graphically displays the decision curve analysis showing the net benefit of using the AMH <4 ng/ml threshold in terms of +SR at mTESE.

## Discussion

Despite the urgent need in terms of everyday clinical practice, the identification of possible markers predicting +SR at mTESE is still a challenge, particularly in men with iNOA. In this context, it becomes pivotal to give the proper indication to surgery in this subcategory of patients, allowing both the patient and the physician to be reasonably confident about the possibility of successful SR at surgery, with the best tailored cost-effectiveness ratio. Yet, a non-negligible proportion of men with iNOA undergo unnecessary mTESE because of an eventual negative SR result that is not foreseeable before the surgery itself. Even though mTESE is considered a reasonably safe procedure, with few long-term sequelae, the surgical exploration of the testis per se may cause intra-, peri-, and postoperative complications (e.g. chronic testicular pain, testosterone deficiency, etc.) (Okada et al., 2002; Ramasamy *et al.*, 2005; Achermann *et al.*, 2021). In this regard, having clinically reliable and user-friendly preoperative parameters to effectively select the appropriate candidates to undergo surgery is certainly a major unmet need. To further support this issue, almost half of our cohort of men with iNOA undergoing mTESE had −SR. To answer this clinical challenge, we strived to identify a potential predictive marker of successful +SR at surgery. To achieve this, apart from descriptive statistics, we used an MVA logistic regression model, which identified serum AMH levels as the only clinical biomarker independently associated with +SR at mTESE (after accounting for several possible clinical confounders) in patients with iNOA.

The predictive role of AMH has been observed in other previous studies. One of the first was conducted by Mitchell *et al.* (2010) on a cohort of 139 men with NOA undergoing mTESE at a single centre. The authors investigated the seminal levels of AMH and InhB in their cohort and concluded that seminal AMH and InhB levels did not differ as a function of TESE outcomes. Their individual and combined receiver operating characteristic curves were below the statistical significance threshold. However, the authors also included men with a diagnosis of Klinefelter syndrome and men with Y microdeletions, which could have largely biased their findings. More recently, Alfano et al. (2017) investigated the role of serum AMH in a more homogenous single-centre cohort of 47 white-European men with iNOA undergoing mTESE; their results showed that while circulating hormone levels associated with a condition of primary hypogonadism did not predict SR, AMH levels, and the AMH/tT ratio did achieve independent predictor status for SR outcomes at mTESE, with a predictive accuracy of 93% and 95%, respectively. Therefore, in the present study we tried to explore the potential predictive role of AMH/tT ratio (Table 2) in our larger cohort, but the AMH/tT ratio did not achieve statistical significance in UVA logistic regression analysis. On the contrary, we confirmed that circulating AMH level was independently associated with +SR. More recently, in a cohort of 155 men with azoospermia, Benderradji *et al.* (2021) demonstrated that AMH can be used as a marker for spermatogenesis in this sub-set of patients with azoospermia; however, the cohort of patients included men with azoospermia (both obstructive and non-obstructive) as well as factors associated with male infertility itself (e.g. cryptorchidism, genetic and karyotype alterations, such as Klinefelter syndrome), which could have biased their analyses. Moreover, the group did not use logistic regression models to explore the predictive role of AMH but rather they showed the differences in terms of AMH levels between the groups considered (Benderradji *et al.*, 2021). In this context, we found in our cohort that those men with +SR had lower E_2_ levels and lower AMH levels compared to those with −SR (Fig. 1). Overall, AMH, together with InhB, the two SCs hormones, are known to regulate genital masculinization and provide negative feedback regulation of FSH secretion, respectively (Salonia *et al.*, 2019, 2021). Following its role during embryogenesis, AMH tends to decrease over time in male individuals. In fact, immature (prepubertal) SCs secrete AMH abundantly until puberty; after puberty AMH starts to decrease (pubertal decline), probably reflecting the maturations of SCs (Grinspon and Rey, 2010; Pietiläinen *et al.*, 2012). As such, our findings could reflect the potential immaturity of SCs among those men with −SR at mTESE; indeed, these men displayed higher levels of preoperative AMH compared with the +SR counterpart. This, in turn, could explain why certain men with iNOA may harbour an even more severe level of azoospermia, with SCs in a more primordial cell state compared to other men with iNOA (Alfano *et al.*, 2021).

Other studies have tried to investigate the predictive role of AMH among men with azoospermia. For instance, Aboukhshaba *et al.* (2021) retrospectively analysed a cohort of 46 men with NOA and concluded that serum AMH was a moderately effective predictor of SR at first mTESE attempt, with high sensitivity and relatively limited specificity. In addition, Renault *et al.* (2022) investigated the predictive factors in a total of 157 non-mosaic 47, XXY Klinefelter syndrome patients undergoing mTESE. The authors found that higher AMH and InhB plasma levels seemed to be related to the presence of foci of spermatogenesis, in which SCs functions are improved, in contact with germ cells with a 46, XY chromosomal complement (Renault *et al.*, 2022). These findings of course are in contrast with ours and other published findings; however, the authors took into consideration a completely different population of men, thus including men with Klinefelter syndrome only. This underlines how important it is, when validating our current findings, to carefully select the study population as some markers could work for some sub-sets of azoospermic patients (e.g. iNOA) but not for others (e.g. Klinefelter syndrome and Y microdeletions).

To the best of our knowledge, this is the first study to investigate several clinically reliable and user-friendly potential predictive factors for SR in a multi-centric homogeneous cohort of men with iNOA undergoing mTESE. As most previous studies have been single centre, with the intrinsic limitation of selection biases, we aimed to reduce this limitation as much as possible by performing a multi-centric study. Moreover, most of the published studies took into consideration a very heterogeneous cohort of azoospermic men (e.g. obstructive azoospermia, Klinefelter syndrome, Y microdeletions, etc.), which could have influenced their findings. Therefore, our study was intentionally designed to include only men with true iNOA to answer a cutting-edge clinical question regarding the selection of who would benefit the most from mTESE surgery and who would not; as we are still far from answering this challenge, we have tried to provide insight towards this.

Likewise, our study is not devoid of limitations. First, although having data only from white-Europeans may only represent a further strength of the analyses, different geographic areas and ethnicity groups might generate different results. Therefore, our findings should be externally validated in even larger cohorts across different centres and ethnic populations. Second, although we did find an interesting association between lower serum AMH levels and a higher likelihood of successful +SR at mTESE among men with iNOA, preoperative AMH levels should not be considered standalone biomarkers to completely obviate the need for mTESE. Rather, AMH levels should be used as a valuable tool to counsel patients about their real chances of success with mTESE, as it can be a useful indicator of patients’ spermatogenesis and SC function. Third, we could not retrieve data regarding the number of spermatozoa that were frozen after a +SR. Systematic reviews and meta-analyses to provide a high level of evidence are lacking in the context of AMH and SR rates in men with iNOA, limiting our ability to reach definite conclusions. Ultimately, future research in this field could explore a range of promising avenues to improve the predictive value of AMH. By identifying additional biomarkers and considering other relevant factors, it may be possible to achieve higher predictive accuracy, better informing patients with iNOA about the potential outcomes of mTESE.

## Conclusion

The lack of reliable biomarkers to predict +SR at mTESE unfortunately makes this procedure unnecessary for a substantial proportion of men with NOA. Overall, almost one in two men with iNOA had a −SR at surgery in our cohort. Overall, men with iNOA with lower circulating AMH levels showed a higher chance of successful SR at mTESE, potentially reflecting a more mature status of their SCs and thus having higher chances of finding spermatogenic foci during surgery. An AMH <4 ng/ml threshold emerged as having good accuracy to positively predict +SR at mTESE. Lastly, current findings certainly do not support a recommendation for using AMH levels as a standalone biomarker during the management work-up of men with iNOA, but rather they should be used as a preoperative counselling tool to better discuss patients’ expected outcomes at the time of mTESE itself.

## Data Availability

The data underlying this article will be shared on reasonable request to the corresponding author.
